# Prevalence and risk factors of burnout among employees at COVID-19 vaccination centers: A cross-sectional study

**DOI:** 10.1371/journal.pone.0322803

**Published:** 2025-05-08

**Authors:** Alaa Mathkour, Abdullah Hassan Alzahrani, Bayapa Reddy Narapureddy, Faris Maeed Alqahtani, Abdullah M. Alshehri, Majed Atiah Althagafi, Shmookh Mohsen Alsyd, Shaik Mohammed Asif, Sunil Kumar Vaddamanu

**Affiliations:** 1 Public Health Administration, Jazan Specialist Hospital, Health Holding Co, Jazan Health Cluster, Jazan, Saudi Arabia; 2 Ministry of Health, Aseer, Strategic Planning Administration, Abha, Saudi Arabia; 3 Department of Public Health, College of Applied Medical Sciences, King Khalid University, Abha, Saudi Arabia; 4 Department of Communicable Diseases Prevention & Ports Administration, Executive Administration of Public Health, Aseer Health Cluster, Health Holding Co, Abha, Saudi Arabia; 5 Preventive Medicine Consultant, Communicable Diseases Department, General Directorate of Health Affairs, Aseer Region, Abha, Saudi Arabia; 6 Public Health Department, Alqari Bani Malik Hospital, Health Holding Co, Taif Health Cluster, Taif, Saudi Arabia; 7 Ministry of Health, Riyadh, Saudi Arabia; 8 Department of Diagnostic Science and Oral Biology, College of Dentistry, King Khalid University, Abha, Aseer Region, Kingdom of Saudi Arabia; 9 Department of Allied Dental Health Sciences, College of Applied Medical Sciences, King Khalid University, Abha, Saudi Arabia; 10 Health and Medical Research Center, King Khalid University, Abha, Saudi Arabia; Virginia Mason Franciscan Health, UNITED STATES OF AMERICA

## Abstract

**Background:**

Health care workers working in Covid-19 vacciantion centers due to their exponential demand experience burnout and stress. Burnout, a psychological syndrome is characterized by emotional exhaustion (EE), depersonalization (DP), and reduced personal accomplishment (PA). It can adversely affect professional and personal well being of an individual. Aim of this study was to check prevalence of burnout among health care workers, to identify personal and work-related factors, and to compare the risk factors associated with the different dimensions of burnout (EE, DP, and PA).

**Methods:**

This cross-sectional study was carried out among 180 employees of various COVID-19 vaccination centers. Three dimensions of burnout (EE, DP, and PA) were evaluated usinge Maslach Burnout Inventory (MBI), and association between burnout and other factors were assessed using logistic regression analysis with 95% confidence intervals (CI).

**Results:**

Prevalence rate of burnout among health care workers was 73.3%. Emotional exhaustion being the highest dimension (38.4%) followed by Depersonalization (30.8%) and personal accomplishment (33.1%). Young employees (<30 years) had significantly higher prevalence of burnout compared to old employees (82.4% vs. 52.4%, p = 0.033). Additionally, employees working more than 8 hours/day (OR = 9.98, p = 0.032) and employess with less than 6 hours of sleep/night (OR = 0.39, p = 0.042) had more likely to experience burnout.

**Conclusion:**

There was an increase prevalence rate of burnout observedamong employees at COVID-19 vaccination centers. There was a significant association between personal and work-related factors such as age, working hours, and sleep patterns. Addressing these factors, particularly by promoting better work-life balance and mental health support, is essential to mitigate burnout and improve employee well-being.

## Introduction

The devastating effect of SARS-COV-2 (COVID-19), which emerged from Wuhan city, has caused bizzare global health crisis which shocked the entire health care system. This prolonged pandemic challenge has taken a toll on psychological health of frontline workers [1]. Burnout syndrome (BS) was considered as the most significant psychological impacts on them during pandemic. This condition first described in 1974 by American psychoanalyst Herbert Freudenberger later refined by Maslach in 1976, highlights the significance of early diagnosis and intervention. BS is prevalent in professionals with high levels of commitment, motivation, and emotional investment. Their professions always place them at high level of intense pressure, leading to physical, emotional, and mental exhaustion [2].

BS is a complex phenomenon causing exhaustion, depersonalization, with decreased personal accomplishment which arise due to chronic emotional and interpersonal stress in professional life [3]. According to World Health Organization’s International Disease Classification (ICD-11) BS is considered as n occupational phenomenon. The widely aaccepted BS model proposed by Maslach and Jackson’s, is characterized by three dimensions: emotional exhaustion (EE), depersonalization (DP), and a reduced sense of personal accomplishment (PA) [4].

Several studies stated that, burnout is common among health care workers and is worsened due to work load exhaustion & personal issues. For instance, research in the United States found that 49% of healthcare workers across various roles reported burnout during the pandemic, driven by workload and fear of exposure [5]. Similarly, a study in Iran noted high emotional exhaustion among frontline healthcare workers, particularly physicians, due to intense job demands [6].COVID-19 pandemic further escalated risk of burn out among them due to excess exposure of life-threatening circumstances, unwanted exposure of pathogens, shift overload, and the decreased autonomy of healthcare professionals [7–10]. Due to which health care workers in intensive-care units and COVID-19 vaccine centers, experienced increase magnitude of burnout.

The choice of COVID-19 vaccination centers as the study group stems from their pivotal role in combating the pandemic through the rapid deployment of vaccination programs. Employees in these centers faced exceptional challenges, including heightened workloads due to the urgent need to vaccinate large populations, frequent interactions with anxious or hesitant individuals, and prolonged exposure to stressful conditions amid an evolving public health crisis. Among these employees, physicians played a central role, overseeing vaccine administration, assessing patient eligibility, and managing adverse reactions, often under intense pressure and extended hours. These factors likely amplified the risk of burnout, a phenomenon already prevalent among healthcare workers but potentially more pronounced in this setting due to the unprecedented demands of the vaccination rollout. Despite this, limited research has explored burnout specifically among vaccination center employees, with studies in China and Malaysia highlighting elevated burnout among vaccination staff due to workload and public-facing roles, yet sparse data exist for regions like the Aseer Region of Saudi Arabia, where the healthcare system encountered significant strain during the pandemic [11,12].

Henceforth, this cross sectional study evaluates the prevalence and predictors of burnout among health care workers working at COVID-19 vaccination centers of Aseer Region, Saudi Arabia, where sparse research has been conducted on the topic [13].

## Methodology

This cross-sectional study was conducted in COVID-19 vaccine centers located in the Aseer Region, southwestern Saudi Arabia. More than 22 vaccine centers were operating in the region during the study period.

The study population comprised all employees working at COVID-19 vaccine centers in Aseer, including doctors, nurses, paramedics, administrative staff, and security personnel. A purposive sampling technique was employed to include the target population.

### Inclusion and exclusion criteria

Participants were included if they were employees actively working at COVID-19 vaccination centers in the Aseer Region during the study period (February 2 to April 15, 2022) and provided informed consent to participate. Employees were excluded if they were not directly involved in vaccination center operations (e.g., external consultants or temporary staff not on regular duty), were on extended leave or vacation for the entire study period, or declined to participate in the study.

### Sample size

A total of 180 employees were invited to participate in the study, with 172 agreeing to take part, yielding a 95.5% response rate. Initially, questionnaires were distributed via email to center directors, but low response rates prompted a shift to distributing questionnaires through private WhatsApp messages to employees.

### Data collection period

Data was collected from 02 February to15 April 2022. Informed consent was taken.

### Data collection methods and instruments

A self-administered Google Forms questionnaire was used to collect data through a website link. Burnout was measured using the Maslach Burnout Inventory for Human Services Survey (MBI-HSS), which consists of 22 items divided into three domains: emotional exhaustion (EE), depersonalization (DP), and personal achievement (PA). Participants rated their responses on a 7-point Likert scale. In addition, the questionnaire included demographic and professional information such as age, gender, job title, and work hours per day.

Due to an initially low response rate from distributing the questionnaire via email to center directors, the research team shifted to using private WhatsApp messages for distribution. The study investigators, affiliated with the research team from King Khalid University and regional health authorities, directly sent the Google Forms link to employees’ personal WhatsApp numbers, which were obtained from vaccination center supervisors with prior permission. This method ensured broader reach among the diverse staff, including doctors, nurses, paramedics, administrative personnel, and security workers.

To maintain subject data confidentiality, no personally identifiable information (e.g., names, phone numbers) was collected within the Google Forms responses. The questionnaire link was shared via WhatsApp with a unique access code for each participant, ensuring responses were anonymous and unlinkable to individual phone numbers. Data transmission was secured through Google Forms’ encryption, and access to the collected data was restricted to the principal investigators, stored on password-protected university servers.

Informed consent was obtained digitally through the Google Forms platform. Before accessing the questionnaire, participants encountered an introductory page explaining the study’s purpose, voluntary nature, and confidentiality measures, followed by a mandatory consent checkbox. Only those who checked “I agree to participate” could proceed to the survey questions. This process was adapted for WhatsApp distribution by including a brief consent overview in the initial message, directing participants to the full consent form within the link.

To accommodate the diverse educational backgrounds of participants (ranging from security personnel to physicians), the questionnaire was designed in simple, clear Arabic and English, with instructions and terms (e.g., Likert scale) explained in lay language. The MBI-HSS items were pre-validated for diverse populations, and demographic questions included an education level option (e.g., high school, diploma, bachelor’s, postgraduate) to capture this variable without assuming literacy barriers. A pilot test with 10 employees from varied roles ensured comprehension before full distribution.

Collected data was stored for a period of two years following the study’s completion (until April 15, 2024) to allow for analysis and potential follow-up, as per institutional guidelines. Data was stored in a de-identified format on secure, encrypted servers at King Khalid University, accessible only to authorized research team members via multi-factor authentication. After this period, the data will be securely deleted unless required for further approved research.

### Data analysis

Data analysis was performed using SPSS version 22.0. The parameters of EE, DP, and PA were categorized into high, average, and low burnout using Maslach’s cutoff values. Chi-square tests were used to explore the relationship between burnout domains and socio-demographic variables, and multiple logistic regression analyses were conducted to identify predictors of burnout. A p-value of less than 0.05 was considered statistically significant.

### Ethical considerations

Ethical approval was obtained from the Asser Institutional Review Board of Directorate Health affairs, Aseer Region (REC-12-01-2022). Consent was obtained digitally as part of the questionnaire process, ensuring that participation was voluntary and informed. Participants received an introductory page via Google Forms outlining the study’s purpose, risks, benefits, and confidentiality measures, followed by a mandatory consent checkbox. Only those who actively agreed could proceed, safeguarding their autonomy and aligning with ethical standards. This approach, adapted for WhatsApp distribution, implies that participants had the opportunity to review and accept the terms before contributing data, minimizing coercion risks in a high-pressure work environment. Confidentiality was ensured, and participants showing signs of burnout were referred to psychiatric services for further support.

## Results

This study included 172 individuals, with 58.1% aged 30–39 years and 52.9% female. Most were Saudi nationals (94.8%) and married (58.7%). A significant portion (50.6%) did not exercise regularly, and 63.4% slept 6–8 hours daily. Notably, 17% reported chronic diseases, with asthma (7.6%) and diabetes (5.8%) being most common. Regarding work-related factors, 63.4% worked in vaccination centers, and nurses constituted 37.8% of the workforce. The majority worked ≤ 8 hours per day (87.2%), with 57% considering leaving their job due to work pressure and other reasons. Burnout was prevalent in 73.3% of participants, with high emotional exhaustion in 38.4%, depersonalization in 30.8%, and low personal accomplishment in 33.1%. Table 1

**Table 1:** Distribution of job schedules among respondents.

	Frequency	Percentage
Average working hours per day		
≤ 8 hours	150	87.2
> 8 hours	22	12.8
Daily rest time (Breaks during shifts)		
No	85	49.4
Yes	87	50.6
Absence or Vacation in the past 6 months		
No	89	51.7
Yes	83	48.3
Reason of absence		
Annual leave	57	33.1
Sick leave	26	15.1

In this study, the overall prevalence of burnout among employees at COVID-19 vaccination centers was measured using the Maslach Burnout Inventory (MBI). The results revealed that 73.3% of the participants exhibited burnout. Emotional exhaustion (EE) was high in 38.4%, depersonalization (DP) was high in 30.8%, and personal accomplishment (PA) was low in 33.1% of participants. This high prevalence may be attributed to the stressful work environment and long working hours during the pandemic. Table 2

**Table 2:** Distribution of respondents in three dimensions of burnout.

Burnout Dimension	Frequency	Percentage
Emotional exhaustion		
Low (≤ 13)	66	38.4
Medium (14–26)	40	23.3
High (≥ 27)	66	38.4
Depersonalization		
Low (≤5)	72	41.9
Medium (6–9)	47	27.3
High (≥10)	53	30.8
Personal accomplishment		
Low (≤33)	57	33.1
Medium (34–39)	43	25.0
High (≥40)	72	41.9
Burnout cases (EE ≥ 27, OR DP ≥ 10 OR PA ≤ 33)		
No	46	26.7
Yes	126	73.3

Personal and work-related factors were identified as significant contributors to burnout. There was a statistically significant association between age and burnout, with younger employees (<30 years) showing a higher prevalence of burnout (82.4%) compared to older employees (≥40 years) at 52.4% (p = 0.033, Table 3). Working hours were also a key factor, as employees working more than 8 hours daily had significantly higher odds of burnout (OR = 9.98, 95% CI: 1.22–81.85, p = 0.032, Table 4). Additionally, participants who slept less than 6 hours per night were more likely to experience burnout compared to those who slept 6–8 hours (OR = 0.39, 95% CI: 0.16–0.97, p = 0.042, Table 4). These findings suggest that prolonged work hours and insufficient sleep are major contributors to burnout.

**Table 3:** Association between personal characteristics and burnout.

			Burnout Cases		P-value
			No	Yes	
Age Groups	<30	No.	9	42	0.033*
		%	17.6%	82.4%	
	30-39	No.	27	73	
		%	27.0%	73.0%	
	≥40	No.	10	11	
		%	47.6%	52.4%	
Gender	Female	No.	26	65	0.566
		%	28.6%	71.4%	
	Male	No.	20	61	
		%	24.7%	75.3%	
Marital Status	Single	No.	11	42	0.090
		%	20.8%	79.2%	
	Married	No.	33	68	
		%	32.7%	67.3%	
	Widowed or divorced	No.	2	16	
		%	11.1%	88.9%	
Living with	Alone	No.	1	9	0.292
		%	10.0%	90.0%	
	With family/relatives or friends	No.	45	117	
		%	27.7%	72.2%	
Income categories (SR)	<5000	No.	8	29	0.120
		%	21.6%	78.4%	
	5000-9999	No.	17	26	
		%	39.5%	60.5%	
	10000-14999	No.	17	49	
		%	25.8%	74.2%	
	≥15000	No.	4	22	
		%	15.4%	74.2%	
Exercise groups	No regular exercise	No.	19	68	0.332
		%	21.8%	78.2%	
	1-4 times	No.	19	42	
		%	31.1%	68.9%	
	5-7 times	No.	8	16	
		%	33.3%	66.7%	
Sleeping hours	< 6 hours	No.	9	46	0.089
		%	16.4%	83.6%	
	6-8 hours	No.	35	74	
		%	32.1%	67.9%	
	> 8 hours	No.	2	6	
		%	25.0%	75.0%	
Smoking Status	Never Smoke	No.	38	100	0.949
		%	27.5%	72.5%	
	Ex-smoker	No.	4	14	
		%	22.2%	77.8%	
	Current smoker	No.	4	12	
		%	25.0%	75.0%	
Chronic illness	No	No.	38	101	0.718
		%	27.3%	72.7%	
	Yes	No.	8	25	
		%	24.2%	75.8%	
Psychological disease	No	No.	44	116	0.519
		%	27.5%	72.5%	
	Yes	No.	2	10	
		%	16.7%	83.3%	

* *Statistically significant*

**Table 4:** Simple and multivariate logistic regression for the factors associated with burnout.

	uOR	95% C.I. for OR		P-value	aOR	95% C.I. for OR		P-value	
Age Group									
<30	1.00				1.00				
30-39	0.58	0.25	1.35	0.205	0.77	0.27	2.16		0.618
≥40	0.24	0.08	0.72	0.011	0.42	0.10	1.83		0.250
Marital Status									
Single	1.00				1.00				
Married	0.54	0.25	1.18	0.123	0.52	0.19	1.44		0.211
Widowed/divorced	2.10	0.42	10.51	0.369	2.73	0.50	14.86		0.246
Income categories (SR)									
<5000	1.00				1.00				
5000-9999	0.42	0.16	1.14	0.089	0.48	0.15	1.52		0.212
10000-14999	0.80	0.31	2.07	0.639	0.88	0.31	2.54		0.818
15000 +	1.52	0.41	5.69	0.537	1.86	0.40	8.62		0.426
Sleeping hours									
< 6 hours	1.00				1.00				
6-8 hours	0.41	0.18	0.94	0.035	0.39	0.16	0.97		0.042
> 8 hours	0.59	0.10	3.39	0.551	0.31	0.05	2.14		0.237
Workplace									
Vaccination Center	1.00				1.00				
Vaccination Clinic	0.60	0.30	1.19	0.140	0.77	0.35	1.72		0.528
Average working hours per day									
≤ 8 hours	1.00				1.00				
> 8 hours	9.00	1.18	68.96	0.034	9.98	1.22	81.85		0.032

When comparing risk factors associated with the different dimensions of burnout, the study found that lack of regular exercise and longer working hours were significantly associated with emotional exhaustion. Participants who did not exercise regularly had higher emotional exhaustion (48.3%) compared to those exercising 5–7 times weekly (25%, p = 0.016, Table 5). Similarly, those working more than 8 hours daily had higher odds of emotional exhaustion (OR = 5.34, 95% CI: 1.65–17.24, p = 0.005, Table 6). Depersonalization was more common among males (43.2%) than females (24.2%, p = 0.046) and those working longer hours Table 5. These findings highlight the importance of addressing both personal and job-related factors, such as sleep, exercise, and work hours, to mitigate burnout among healthcare workers. Table 6.

**Table 5:** Association between personal characteristics and Emotional Exhaustion.

			Emotional Exhaustion		P-value
			Low/Moderate	High	
Age Groups	<30	No.	30	21	0.166
		%	58.8%	41.2%	
	30-39	No.	60	40	
		%	60%	40%	
	≥40	No.	17	4	
		%	81.0%	19.0%	
Gender	Female	No.	61	30	0.167
		%	76.0%	33%	
	Male	No.	46	35	
		%	56.8%	43.2%	
Marital Status	Single	No.	32	21	0.892
		%	60.4%	39.6%	
	Married	No.	63	38	
		%	62.4%	37.6%	
	Widowed or divorced	No.	12	6	
		%	66.7%	33.3%	
Living with	Alone	No.	7	3	0.744
		%	70.0%	30.0%	
	With family/ relatives or friends	No.	100	62	
		%	61.7%	38.3%	
Income categories	Less than 5000	No.	25	12	0.776
		%	76.6%	32.4%	
	5000-9999	No.	28	15	
		%	65.1%	34.9%	
	10000-14999	No.	39	27	
		%	59.1%	40.9%	
	≥ 15000	No.	15	11	
		%	57.7%	42.3%	
Exercise groups	No regular exercise	No.	45	42	0.016*
		%	51.7%	48.3%	
	1-4 times	No.	44	17	
		%	72.1%	27.9%	
	5-7 times	No.	18	6	
		%	75%	25%	
Sleeping hours	< 6 hours	No.	29	26	0.041*
		%	52.7%	47.3%	
	6-8 hours	No.	75	34	
		%	68.8%	31.2%	
	> 8 hours	No.	3	5	
		%	37.5%	62.5%	
Smoking Status	Never Smoke	No.	85	53	0.916
		%	61.6%	38.4%	
	Ex-smoker	No.	12	6	
		%	66.7%	33.3%	
	Current smoker	No.	10	6	
		%	62.5%	37.5%	
Chronic illness	No	No.	87	52	0.833
		%	62.6%	37.4%	
	Yes	No.	20	13	
		%	60.6%	39.4%	
Psychological disease	No	No.	102	58	0.215
		%	63.7%	36.3%	
	Yes	No.	5	7	
		%	41.7%	58.3%	

* *Statistically significant*

**Table 6:** Association between Professional Characteristics and Depersonalization.

			Depersonalization		P-value
			Low/Moderate	High	
Workplace categories	Vaccination Center	No.	73	36	0.408
		%	67.0%	33.0%	
	Vaccination Clinic	No.	46	17	
		%	73.0%	27.0%	
Job groups	Administrative	No.	18	10	0.010*
		%	64.3%	35.7%	
	Lab specialist or pharmacist	No.	5	5	
		%	50.0%	50.0%	
	Nurse	No.	51	14	
		%	78.5%	21.5%	
	Other	No.	18	6	
		%	75.0%	25.0%	
	Physician	No.	7	12	
		%	36.8%	63.2%	
	Public health specialist	No.	9	1	
		%	90.0%	10.0%	
	Security	No.	11	5	
		%	68.8%	31.3%	
Duration of Work in the Vaccination Center/Clinic (in months)	<6 months	No.	33	16	0.929
		%	67.3%	32.7%	
	6-11 months	No.	77	34	
		%	69.4%	30.6%	
	≥12 months	No.	9	3	
		%	75.0%	25.0%	
Employee at MOH	No	No.	18	6	0.506
		%	75%	25%	
	Yes	No.	101	47	
		%	68.2%	31.8%	
Average Number of clients per day	≤ 20	No.	7	4	0.739
		%	63.6%	36.4%	
	> 20	No.	112	49	
		%	69.6%	30.4%	
Work Schedule	Morning Shift	No.	78	31	0.581
		%	71.6%	28.4%	
	Night Shift	No.	27	13	
		%	67.5%	32.5%	
	Rotating Shift	No.	14	9	
		%	60.9%	39.1%	
Average working hours per day	≤ 8 hours	No.	110	40	0.002*
		%	73.3%	26.7%	
	> 8 hours	No.	9	13	
		%	40.9%	59.1%	
Weekly holiday	I don’t have a weekend	No.	2	4	0.171
		%	33.3%	66.7%	
	1 day/week	No.	21	10	
		%	67.7%	32.3%	
	2 day/week	No.	96	39	
		%	71.1%	28.9%	
Daily Rest time	No	No.	59	26	0.949
		%	69.4%	30.6%	
	Yes	No.	60	27	
		%	69.0%	31.0%	
Absence from work	No	No.	67	22	0.073
		%	75.3%	24.7%	
	Yes	No.	52	31	
		%	62.7%	37.3%	
Thinking of leaving the COVID-19 Vaccine Center	No	No.	74	0	< 0.001*
		%	100%	0%	
	Yes	No.	45	53	
		%	45.9%	54.1%	

* *Statistically significant*

## Discussion

The study identified a high burnout prevalence (73.3%) among employees in COVID-19 vaccination centers, consistent with global reports showing elevated burnout among healthcare workers during the pandemic. The findings align with other studies, such as Alwashmi AH et al. [14], which reported similar rates of burnout among healthcare professionals in Saudi Arabia. However, the variation in burnout rates across different studies highlights the impact of cultural factors, healthcare systems, and pandemic stressors.

The results indicate a burnout prevalence of 73.3% among vaccination center employees, a high rate that mirrors findings from other pandemic-related healthcare studies. For instance, Dimitriu MCT et al. [15] reported a burnout prevalence of 76% among Romanian medical residents, while Alwashmi AH et al. [14] found that 80.2% of physiatrists in Saudi Arabia experienced burnout. However, our study’s burnout rate is significantly higher than the 20.57% reported by Konlan KD et al. [3] among healthcare workers in Ghana, highlighting the intense stressors unique to employees in COVID-19 vaccination centers during the pandemic.

Key personal and work-related factors were identified as significant predictors of burnout. Among these, working hours played a crucial role. Employees working more than 8 hours daily had a higher likelihood of experiencing emotional exhaustion and depersonalization. This is consistent with previous studies, such as that of Elghazally SA et al. [16], which found that extended work hours increased emotional exhaustion and depersonalization in healthcare professionals. Similarly, sleep deprivation emerged as a critical factor in this study. Employees who reported getting less than 6 hours of sleep per night were more likely to suffer from burnout. This aligns with findings by Giorgi F et al. [17] and Ibnshamsah S et al. [18], who noted the relationship between poor sleep quality, increased job strain, and burnout. Adequate sleep appears to offer a protective effect against the mental and physical toll of burnout, reinforcing the importance of addressing sleep quality in high-stress healthcare environments.

Factors such as insufficient sleep (<6 hours) and working >8 hours daily were strongly associated with burnout. This mirrors the findings by Ibnshamsah S et al. [18] and Chin W et al. (2021), [19] who noted a clear relationship between limited sleep and increased burnout among healthcare workers. Moreover, longer working hours have consistently been shown to exacerbate burnout, as supported by studies in both local Chin W et al., [19] and international contexts.

In terms of emotional exhaustion, participants who exercised regularly and worked fewer hours reported lower levels of exhaustion. This is in line with studies like Wang H et al. [20] and Naczenski LM et al. [21], which emphasize the protective role of physical activity in reducing emotional exhaustion and stress. Sports participation helps healthcare workers cope with stress, potentially improving their mental well-being.

Physical exercise also played a significant role in mitigating emotional exhaustion. Employees who engaged in physical activity 1–4 times weekly reported lower odds of experiencing emotional exhaustion, a result consistent with Wang J et al., who found that regular exercise reduces stress and improves emotional well-being among healthcare workers. Naczenski LM et al. [21] also identified physical exercise as a key intervention for reducing emotional exhaustion in systematic reviews. This finding highlights the potential benefit of promoting physical activity as part of burnout prevention programs in healthcare settings.

Depersonalization, another key component of burnout, was associated with extended work hours and higher professional demands, particularly among physicians. This trend has been documented in various studies across the Middle East, where physicians often face heavy workloads, leading to higher depersonalization rates Radwan MZ et al., [22] Asghar MS et al., [23].

Employees working more than 8 hours daily had higher odds of depersonalization, echoing findings by Radwan et al.[22] and Shorub E et al. and Thorsen VC et al. [24,25], who found that extended working hours, particularly during the COVID-19 pandemic, were linked to increased feelings of detachment and cynicism among healthcare workers [26].

## Conclusion

The study highlights the critical issue of burnout among employees in COVID-19 vaccination centers, driven by factors such as excessive work hours, lack of sleep, and insufficient exercise.

To decrease burnout among this population, the authors suggest the following concrete measures based on the study results: (1) Implement mandatory shift limits to ensure no employee works more than 8 hours per day, given the significantly higher odds of burnout (OR = 9.98, p = 0.032) with extended hours; (2) Establish a workplace policy promoting at least 6 hours of sleep per night, such as providing rest areas or staggered shifts, as insufficient sleep increased burnout risk (OR = 0.39, p = 0.042); (3) Introduce a subsidized exercise program, encouraging 1–4 weekly sessions, which reduced emotional exhaustion (p = 0.016) in this study; and (4) Provide on-site mental health support, including regular screenings and counseling, to address the high prevalence of burnout (73.3%). These findings suggest that targeted interventions addressing these risk factors could significantly alleviate burnout. Healthcare systems should prioritize addressing these risk factors to enhance worker well-being and reduce attrition rates, ensuring better patient care during crises like the COVID-19 pandemic.

## Supporting information

S1 Data Final data. (XLSX)
